# The Importance of Derivatizing Reagent in Chromatography Applications for Biogenic Amine Detection in Food and Beverages

**DOI:** 10.1155/2020/5814389

**Published:** 2020-01-02

**Authors:** Muhammad Abdurrahman Munir, Khairiahi Haji Badri

**Affiliations:** Faculty of Science and Technology, Universiti Kebangsaan Malaysia, Bangi, Selangor, Malaysia

## Abstract

Biogenic amines (BA) are chemical compounds formed in foods that contain protein, allowing the foods to undergo a bacterial degradation process. Biogenic amines are labeled as toxic food because its consumption exceeding the FDA regulation (50 mg/kg) can be harmful to humans. Some countries also have regulations that prohibit the consumption of biogenic amines in high concentrations, especially histamine. The chromatography methods generally applied by researchers are liquid chromatography (LC) and gas chromatography (GC), where the use of a derivatization reagent is necessary to increase their sensitivity. This review is based on past and present studies about biogenic amine detection related to food samples. The rationale of this study is also to provide data on the comparison of the analytical approaches between LC and GC methods. Furthermore, the various approaches of biogenic amine determination and the most applied analytical methods have been reviewed.

## 1. Introduction

Food analysis has become a challenge in the food industry in ensuring the food distributed is safe for human consumption. There are many factors that require foods to be analyzed, such as contamination of food by bacteria, mishandling of food by humans, and food degradation due to environmental factors such as pH, temperature, and storage. Biogenic amines are compounds that can be easily found in food protein and should be analyzed because they can bring adverse effects. The appearance of biogenic amines in foods is generally influenced by bacteria contamination [1]. The review paper describes the importance of biogenic amine detection and the methods applied by some studies for biogenic amine detection in food and beverage samples.

## 2. Biogenic Amines

**Figure 1:**
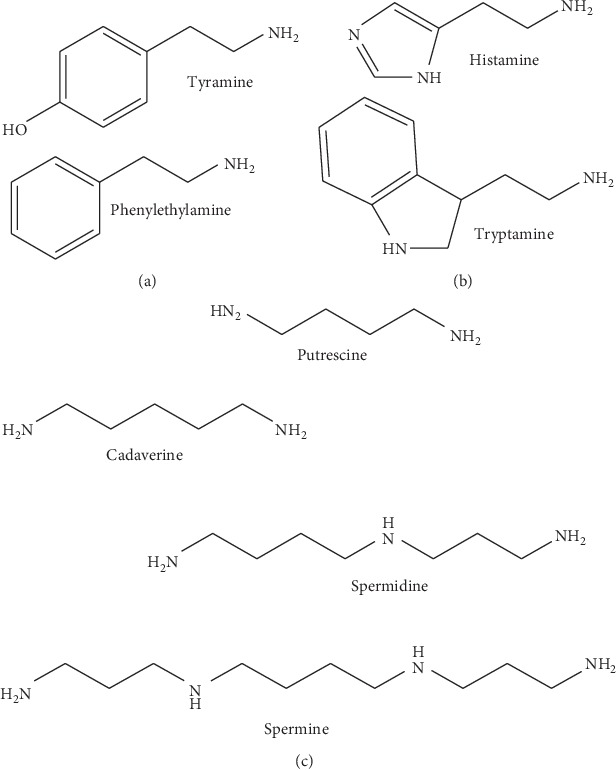
The main biogenic amines' chemical structures found in food and beverages ([4] with modifications): (a) aromatic structure; (b) heterocyclic structure; (c) aliphatic structure.

Biogenic amines (BA) are organic compounds with aliphatic, aromatic, and heterocyclic structures and formed by amino acid decarboxylation. The alpha-carboxyl group is removed from the amino acid compound causing the production of the following biogenic amines: histamine from histidine, cadaverine from lysine, tyramine from tyrosine, tryptamine from tryptophan, etc. [2, 3].  Figure 1 shows several biogenic amines that are generally found in food. Of all the biogenic amines, histamine is particularly found in the human body, albeit at a low and harmless concentration owing to the presence of diamine oxidase (DAO) and histamine-N-methyltransferase (HMT) to detoxify histamine. Histamine has the ability to increase blood capillary permeability as a side effect of an inflammatory response, while cadaverine can modify histamine from being nontoxic to toxic [5–7]. Foods contaminated with other compounds are safe for consumption after a heating process. Biogenic amines, on the contrary, can withstand heat and are not destroyed by high heat [8, 9].

However, bluefish, mahi-mahi, herring, and sardine known as nonscombroid fish species can also cause histamine poisoning [10]. Several organizations have different perspectives about histamine consumption. The Food and Drug Administration (FDA) regulation for histamine consumption allows histamine to be consumed at 5 mg/100 g of food. Meanwhile, the European Community released a guideline for histamine content in foods and beverages to be lower than 10 mg/100 g of food. However, several studies showed histamine was given to humans orally at 67 and 180 mg per 100 g food without any sign of toxicity [9, 11]. Based on the issues presented above, it is imperative for the players of the food industry to monitor and measure the presence of biogenic amines in food, particularly histamine, to ensure food safety. Food researchers should take on the task of controlling and finding the best method to detect biogenic amines in food and beverages before distribution.

## 3. Biogenic Amine Detection by Chromatography Methods

Biogenic amine detection in foodstuff is crucial for the following reasons: to modify the current technique or develop new techniques to be applied by researchers or analysts in analyzing biogenic amines in various products from other countries that consume food protein regularly; to study the relationship between biogenic amine accumulation and the growth of bacteria inside foods; and to study their potential toxicity [12]. The analysis of biogenic amines in foods is not a straightforward process as biogenic amines' structures are very complex, and their presence in foods is sometimes very small (below 1 ppm). The appropriate extraction and purification methods must be selected with care [13]. Furthermore, to ensure the isolation of biogenic amines, the food samples must be treated very carefully. Sample clean-up is important in a biogenic amine extraction before an analysis using chromatography techniques is carried out. Common clean-up techniques applied are presented in Table 1. These include solid-phase microextraction (SPME), hollow fibre liquid-phase microextraction (HF-LPME), liquid-liquid extraction (LLE), and solid-phase extraction (SPE). Upon completion of a clean-up, the selected instruments can be used to perform an analysis. The use of solvents is important to ensure the successful extraction of biogenic amines. Some studies applied water and methanol to extract biogenic amines, but some studies also considered the use of acidic solvents such as perchloric acid, hydrochloric acid (HCl), or trichloroacetic acid (TCA) for their satisfactory accuracy and recovery.

Several studies reported the use of solvents for biogenic amine extraction, but all of them acquired different results. Biogenic amines are strong organic bases, and thus it is very important to understand their characteristics. 5% TCA and perchloric acid showed satisfactory recovery of biogenic amines and are strongly recommended to extract biogenic amines from fish and meat [16]. Different solvents studied by Richard et al. [17] such as methanol:distilled water (75 : 25, v/v), methanol:0.4 N HCl (75 : 25, v/v), and methanol:HCl:KCl (75 : 24 : 1, v/v/v) were used to extract biogenic amines from fish muscles. The study reported that methanol:0.4 N HCl (75 : 25, v/v) showed good recovery for histamine, putrescine, and cadaverine. Hydrochloric acid 0.1 M is the best choice for amine extraction from cheese but unsuitable for fish and meat because the content of biogenic amines in fish and meat are higher than that in cheese. A study evaluated the application of four acids to extract biogenic amines from cheese, such as chloric acid, perchloric acid, sulfosalicylic acid, and trichloroacetic acid, and reported that perchloric acid has the ability to extract biogenic amines 30% higher than other acid solvents [18]. The use of 0.1 M HCl is preferred to extract biogenic amines from food samples because it is simple and inexpensive. After extraction using HCl, the solution should be alkalinized using sodium or potassium hydroxide (NaOH or KOH) to acquire a good derivatization reaction. A stable pH is necessary for biogenic amine recoveries, where the ideal pH is 11.5. Anything above pH 11.5 reduces the recovery of tyramine. Thus, pH 10 is considered to obtain a good recovery for tyramine. However, it is unstable for histamine, cadaverine, putrescine, spermine, and spermidine [3].

Matrix solid-phase dispersion (MPSD) and solid-phase extraction (SPE) have been applied to extract biogenic amines from various foods [19, 20]. These methods are suitable for fish and meat samples that contain many compounds besides biogenic amines because they can reduce the time of biogenic amine extraction. SPE is used to clean-up fish or meat samples to isolate the specific analyte before the samples are analyzed using chromatographic methods or other instruments, whereas MPSD is used to purify biogenic amines from solid samples such as fish and meat. Furthermore, these techniques require a smaller sample size compared with conventional extraction and use less organic solvents [21]. The liquid chromatography method using C18 column sorbent is used after SPE method is applied to clean-up the analyte. After the samples are derivatized, they are loaded to an activated sorbent, washed, and eluted with acetonitrile for LC analysis [22]. Otherwise, the pH of biogenic amines must be modified to pH 11, and the analytes are eluted and derivatized [19].

Until now, several analytical techniques for biogenic amine detection in food samples have been studied, such as capillary zone electrophoresis (CZE), thin-layer chromatography (TLC), high-performance liquid chromatography (LC), and gas chromatography (GC). The studies showed successes in various samples such as seafood, fish and fish products, juice, milk, dairy products, meat and meat products, and wine [23, 24]. The common chromatography techniques used are high-performance liquid chromatography (HPLC) [24, 25] and gas chromatography (GC) [26, 27].

### 3.1. High-Performance Liquid Chromatography (HPLC)

**Figure 2:**
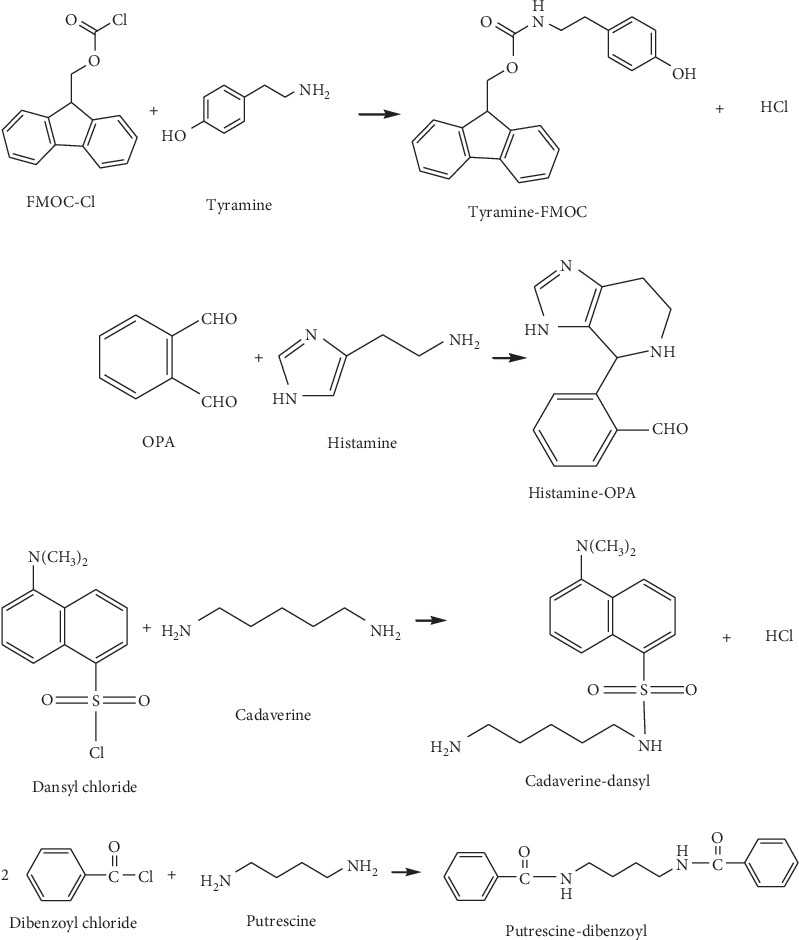
Reaction of derivative reagents such as FMOC-Cl with tyramine, OPA with histamine, dansyl chloride with cadaverine, and dibenzoyl chloride with putrescine, adopted from Lazaro and Conte-Junior [3].

HPLC is the most common chromatography method applied by several studies mainly due to its simplicity and low cost. C8 or C18 is the best column to analyze biogenic amines in samples with mobile phase applied such as acetonitrile, methanol, or deionized water [28]. Biogenic amine analysis using HPLC needs to be labeled by a derivatizing reagent because biogenic amines have low volatility and lack chromophores. As such, derivatization is important to modify their characteristics. Derivatization is divided into two steps, namely, pre- and postcolumn. The use of various chemical reagents during derivatization relies on the HPLC detector used. O-Phthaldialdehyde (OPA), dansyl-, benzoyl- and dabsyl-chloride, fluorescein, and 9-fluorenylmethyl chloroformate (FMOC) [25], and their reactions to biogenic amines are shown in Figure 2. These derivatizing reagents are commonly used by researchers. Some of them are suitable in an ultraviolet (UV) detector, and some are suitable in a fluorescence detector (FD).

The common derivatizing reagents used are OPA, benzoyl chloride, and dansyl chloride. Some researchers use OPA because it is fast and derivatization can be completed below 2 min with a mixture of borate buffer (pH 6–8) and methanol at ambient temperature. It can also react with primary amines easily below 1 min with the addition of a reducing reagent, such as 2-mercaptoethanol or N-acetylcysteine to modify the characteristics of polyamines, such as putrescine, cadaverine, spermidine, and spermine. Nevertheless, some studies reported that OPA has stability issues and a short life [3]. As such, the sensitivity of amine derivatized by OPA during HPLC analysis can decrease. Thus, researchers choose dansyl chloride or benzoyl chloride over OPA as they are more stable. The use of dansyl chloride is suggested for the derivatization step due to its stability for di- and polyamines. Some studies also showed the use of dansyl chloride to detect histamine using the HPLC method with C18 and equipped UV detector with 254 nm providing satisfactory results [29–32]. Nevertheless, dansyl chloride can react with other compounds besides amines such as phenols and alcohols owing to it being a nonspecific reagent. The reaction occurs because food samples contain several hydroxylic substances linked with biogenic amines [33–35]. Thus, several peak interferences will appear besides biogenic amines, and in order to tackle this issue, sorbent particles with a high resolution of 2 *μ*m need to be applied. The separation of 31 compounds of amino acids and biogenic amines using liquid chromatography can be completed within 25 min [36]. On the contrary, the advantage of benzoyl chloride is the long elution time with dansyl derivatives. Procedures using benzoyl chloride were described for several foods such as cheese, fish, and chicken meat by Lázaro et al. [37–39] and Rodrigues et al. [40], respectively, with satisfactory results. Tahmouzi et al. [41] reported the comparison between OPA and benzoyl chloride for histamine determination in tuna fish and found that the derivatization using benzoyl chloride is longer and more complex, but the histamine derivative is more stable than OPA. Nevertheless, the use of derivatization can be avoided using reverse phase columns equipped with an amperometric detector. This technique requires a small sample size and gives better limit detection compared with detectors [42]. The application of benzoyl chloride can also be used to overcome the use of liquid chromatography equipped with double mass spectrometry. As a result of coelution of matrix substances, isotope-labeled internal standard-based methods have been recommended [43].

Almost all isotope-labeled biogenic amines are unavailable as their synthesis is labor intensive. Alternatively, the derivatization of amines with isotope-coded derivatization reagent using 10-methylacridone-2-sulphonyl chloride, and its unlabeled form was recommended [44]. Benzoyl chloride or benzoylation is fast acting and needs to be vortexed at room temperature in alkaline medium for one minute, or inside a borate buffer of pH 10. Nevertheless, the mixture must be kept for 10 to 30 min to allow hydrolysis of the excess reagent. Benzoylation reacts at primary and secondary amine groups, yet tyramine from phenolic groups also reacts with benzoylation [45, 46].

In an alkaline medium for instance, benzoylation can react with ammonium ion, alcohols, polypeptides, and polyphenols. However, benzoylation will adjust the excess of reagent to complete the derivatization step. An experiment has been conducted to find the factors that affect the benzoylation result and optimize the derivatization process, and it was found that the amount of reagent and alkali concentration affects benzoylation yield. Effective mixing of the reaction mixture was necessary to avoid interference or split peaks. Moreover, derivatives in methanol that contain benzoyl chloride must be kept for 3 days at 4°C, to prevent the derivatives from being destroyed [47, 48].

The use of detectors are strongly important after biogenic amines are derivatized with a derivatizing reagent to analyze biogenic amines in food samples. UV and fluorescence detectors (FD) are generally applied, where FD shows better sensitivity than UV. Nevertheless, some studies equipped the HPLC with mass spectrometry (MS) because MS can identify the structure of biogenic amines directly, and derivatization is unnecessary. However, the use of MS to analyze biogenic amines in foods is not favored because UV and FD are less expensive, less time consuming, and are sufficient for biogenic amine detection.  Table 2 summarizes the HPLC methods used to determine biogenic amines in food samples in the last decades.

Based on the table, biogenic amines can be found in solid and liquid samples after extracted by different extraction methods, and all of them are extracted using acid solvents such as TCA and HCl. Histamine, putrescine, and cadaverine are amines that generally can be found in food samples according to the studies, where some of them found more than FDA regulations which we as researchers should be alert about this situation owing to the effects that will be given by biogenic amines. Almost all studies used C18 as their column because the column has capability to separate and detect the samples during the process of analysis The choice of derivatizing agent also influenced the choice of detector. The use of OPA should be followed by the fluorescence detector, whereas the use of dansyl, dabsyl, and benzoyl chloride were suitable for UV detectors. Various wavelengths also applied by some studies where the choice of wavelength influenced by the column, derivatization reagent, and detector.

### 3.2. Gas Chromatography (GC)

Gas chromatography is the general method applied by researchers after HPLC. It has several advantages, such as more accurate, sensitive, and selective, with a higher resolution compared with HPLC. Nevertheless, it is similar to HPLC, where derivatization step is required as biogenic amines have a high polarity and are not easily vaporized, whereas samples need to be vaporized during a GC analysis. Furthermore, solid-phase microextraction (SPME) is a convenient method to extract biogenic amines from samples to obtain a good recovery during a GC analysis. This technique reduces the use of sample preparation, thus reducing sample contamination. Nevertheless, the amine structure influences the sensitivity of GC where the primary amine is more difficult than the secondary, and the secondary amine is more difficult than the tertiary [61]. Application of GC becomes a choice when biogenic amine compounds convert from polar to nonpolar derivatized compounds. The derivatization technique is used to decrease the polarity of amines and modify their characteristic to be chromatographed on GC columns [62–64]. Derivatizing reagents for gas chromatography are divided into three major reagents, namely, alkylation, acylation, and silylation which are elaborated in Table 3. These derivatizing agents are also considered and are suitable for biogenic amines as they can increase the sensitivity of biogenic amines, so they can be easily detected using GC [67]. However, the use of GC detectors has an important role to enhance the sensitivity of GC to detect biogenic amines in various samples. For example, the use of GC equipped with mass spectrometry (MS) combined with a mass selective detector (MSD) that uses a selected ion monitoring (SIM) method can acquire structure information for the identification of specific amines. These detectors provide an advantage where limits below a microgram can be detected. Furthermore, the flame ionization detector (FID) and flame thermionic detector (FTD) provide satisfactory selectivity and sensitivity in detecting amines. The use of flame photometric detector (FPD) and electron capture detector (ECD) can also be used to detect amines, but the derivatization step is required as described above [68, 69]. Several studies using GC for biogenic amine determination in solid and liquid samples are presented in Table 4.

Silylation has the ability to decrease surface adsorption and increase analyte volatility. A silylation agent is used to react with components containing -OH, -SH, and -NH groups producing silyl derivatives which are less polar, more volatile, and thermally more stable. The choice of a silylation reagent relies on the reactivity of compound where sterically crowded reagents with bulkier R groups are less reactive yet are able to produce stable derivatives after using silylation reagent. Based on Table 3, the silylation reagent TMS (trimethylsilyl) is the best option for GC detection. This is because TMS group influences the volatility of analyte for GC and GC-MS applications and contributes to chemical and thermal stability [66]. The use of silylation requires a heating process to accelerate the reaction with analyte; therefore, the use of glassware during derivatization process is compulsory. The reagent contains moisture; and its reaction with water results in the reagent's removal from the reaction with analyte. As such, it is suggested that excess derivatization reagent is used to minimize the moisture content of the reagent, avoiding the use of solvents containing active hydrogen atoms [77]. It is important to isolate and determine the content of biogenic amines from food and beverage samples before they undergo a derivatization process and GC analysis. Distilled water can be used to extract biogenic amines from fish samples due to the solubility properties of biogenic amines. Nevertheless, according to some studies, the use of acidic solvents such as hydrochloric acid and trichloroacetic acid provides better results. Some studies preferred the use of a 5-column GC analysis and MS detector to identify the structure of biogenic amines, with only one study using FID as a detector. The application of silylation is recommended for mass spectrometry applications where the silyl group produces more diagnostic fragments or ions of a particular characteristic used for selected ion monitoring (SIM). The application of a double mass spectrometry or MS/MS detector will not require a derivatization process because MS/MS is extremely sensitive and can detect biogenic amines in food samples. The use of other detectors on the contrary requires the derivatization process to alter the properties of analytes, such as decreasing polarities and increasing volatility of biogenic amines, and improve the selectivity, sensitivity, and resolution of GC analysis.

**Figure 3:**
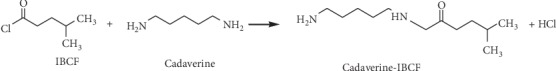
Reaction process for cadaverine derivatized by isobutyl chloroformate (IBCF).

However, the use of MS/MS is very expensive and takes too much time in preparing the sample before analysis [67]. Nevertheless, the use of derivatization agents comes with the following issues: loss of the analytes; occurrence of byproducts; extension of sample preparation time; and potentially longer process [75]. Acylation reagents have become an alternative to silylation reagents for their capability to decrease the polarity of amine, which aids in decreasing nonspecific adsorption effects and modifying GC properties. These reagents also improve stability of compounds by protecting unstable groupings. Furthermore, they confer volatility on substances such as amines which have many polar groups that are nonvolatile and will decompose during heating process, making it possible to detect the amine compounds using gas chromatography. Some studies reported that an acylated compound is more stable than a silylated one, especially for primary amines. Petrarca et al. [70] reported the use of acylation reagent heptafluorobutyric anhydride (HFBA) to detect spermidine and spermine in baby food products using gas chromatography equipped with mass spectrometry. The use of these derivatizing agents provides good accuracy and recovery (above 100%) as the GC with FID and MS detectors is able to detect the derivatized biogenic amines. Successful biogenic amine analysis has also been reported using GC coupled with mass spectrometry (GC-MS) [74, 76]. Besides these derivatizing reagents, Papageorgiou et al. [75] also introduced the use of isobutyl chloroformate (IBCF) to successfully derivatize the structure of amines, as shown in Figure 3.

Shin et al. [78] reported that amines can also be found in human urine besides food and beverages. The amines found in human urine are dopamine, tyramine, and octopamine. The amines in urine samples were extracted using the 0.1 M HCl, derivatized with N-methyl-N-hexamethyldisilazane (HMDS), and modified using N-methyl-bis (heptafluorobutyramide) (MBHFBA). The GC analysis using the MS/MS detector was applied and equipped with DB-5 MS capillary column. Almost all solid and liquid samples contained biogenic amines with half of them containing histamine. Based on the research reported, none of them contained more than 200 ppm of histamine. Although the histamine concentration in the samples was above the FDA regulation (50 ppm), no cases of histamine poisoning were reported in the study.

Based on Table 4, it can be concluded that the addition of derivatizing reagent for GC analysis is imperative to improve the properties of analytes and improve the sensitivity and selectivity of GC. The use of GC (column: ZB-5 MS and column: HP-5 MS) is also suitable to detect biogenic amines in food or beverages. These methods are well approved.

## 4. Conclusion

The characteristics of biogenic amines such as high polarity, stability of compounds, and matrix complexity are the major concerns in the detection of biogenic amines using liquid and gas chromatography techniques. The availability of several methods to extract biogenic amines from food and beverage samples and the choice of specific column for separations in liquid and gas chromatography are fascinating topics. The use of derivatizing reagents during HPLC and GC applications has been developed well by whole studies linked to biogenic amine detection which shows the importance of a derivatization process before biogenic amines are analyzed using HPLC and GC. However, the determination of biogenic amines poses the following issues: product instability; choice of derivatizing reagent; formation of byproducts; and side effect of excess reagent that causes a decrease in the sensitivity of column.

The derivatizing reagent for HPLC analysis, dansyl chloride, becomes the most well-known reagent used for food analysis despite its disadvantages, such as slow reaction, the recovery of derivative being influenced by pH reagent, and formation of byproducts. *o*-Phthalaldehyde is a reagent used to derivatize biogenic amines that contain primary amine groups, whereas FMOC-Cl is a reagent that is suitable for derivatizing compounds that contain secondary amine groups and for increasing the sensitivity of liquid chromatography. In a GC analysis, the most popular reagent for food analysis is silylation, despite several inconveniences associated with its use, such as the need for a heating process and its moisture causing reagent to disappear from the reaction.

Some studies also reported that the use of a double MS detector does not require the derivatization step owing to the MS detector being much more sensitive compared with UV and fluorescence detectors. Furthermore, there are some analytical disadvantages to using derivatization agents and conventional methods such as HPLC and GC. They are not only time consuming but also very expensive and need to be analyzed by experts. Improper derivatization step can also cause incomplete derivatization where the analyte cannot react properly with the derivatizing reagent. Some derivatizing reagents must also be prepared one day before analysis as the reagents can be degraded by temperature and the environment. It shows that derivatizing reagent should be prepared by people with analytical chemistry background. Nevertheless, the determination of biogenic amines needs to be done because some biogenic amines such as histamine, cadaverine, putrescine, and tyramine are toxic if consumed by humans. Thus, researchers should study about derivatizing reagents that are stable, easily prepared, and can be applied in different detectors.

## Figures and Tables

**Table 1:** The different between solid-phase extraction (SPE) and matrix solid-phase dispersion (MPSD) [14, 15].

No.	SPE technique	MPSD technique
1	A method used to purify analytes from a sample by sorption onto a disposable solid-phase cartridge, followed by elution of the analyte with a solvent before analysis using liquid chromatography; this method can increase the selectivity of LC	A method that involves simultaneous disruption and extraction of various solid and semisolid samples owing to the direct mechanical blending of the sample

2	Sample should be prepared step-by-step, and some of the sample components should be dispersed during the process in order to make sure the analyte is suitable for SPE column	This technique makes sure all the sample components are extracted, and none of them is discarded

3	Only the specific analyte will be detected	As none of the components are discarded, physical and chemical interactions inside the components occur

4	Sample is absorbed onto the top of the column packing material	This technique ensures the sample is not absorbed throughout the column

**Table 2:** Various studies based on HPLC techniques for biogenic amine detection in food and beverages.

Food sample	Analyte	Extraction	Stationary phase	Derivatizing/detector	Ref.
Meat and meat products	His, Tyr, Phm, Put, Cad, Trp, Agm, Spr, Spd	7.5% TCA	Cation-exchange	OPA/Fluorescence (ex: 330 nm; em: 465 nm)	Triki et al. [49]
Fermented meat	His, Spr, Spd, Cad, Put, Phm, Tyr, Trp	0.4 M HClO_4_	C18	Dbs-Cl/UV (450 nm)	De Mey et al. [50]
Chicken meat	Tyr, Put, Cad, Spd, Spr	5% HClO_4_	C18	Bnz-Cl/DAD (198 nm)	Lazaro et al. [37–39]
Fish products	His, Put, Tyr	0.4 M HClO_4_	ODS2 150A column	Dns-Cl/PDA (254 nm)	Bilgin and Genccelep [51]
Fish meat	Put, Cad	5% HClO_4_	C18	Bnz-Cl/UV (198 nm)	Rodrigues et al. [41]
Fish meat	His, Spr, Spd, Cad, Put, Tyr, Phm, Trp	5% TCA	C18	LC-ESI-MS/MS	Sagratini et al. [24]
Cheese	His, Cad, Spr, Tryp, Hex	UA-DLLME	C18	BCEC-Cl/FLD	Wu et al. [52]
Cheese	His, Spr, Spd, Put, Tyr, Cad, Agm, Phm	SPE	Cation-exchange	No derivative step/ELSD	Spizzirri et al. [20]
Corn oil	His, Put, Tyr, Cad, Tryp, Spr, Spd	Solvent extraction	C18	Dns-Cl/UV-visible	Yoon et al. [53]
Chocolate, vegetables, and fruits	Tyr	Solvent extraction	RP-18e	No derivatizing/FLD-DAD	Baranowska and Plonka [54]
Fresh milk and fish	His	6% TCA	Reversed-phase 18	Dns-Cl/UV-visible	Arulkumar et al. [55]
Soy sauce	His, Spd, Put, Tyr, Trp, Spr	Acetonitrile	C18	HPLC-MS/MS	Dong and Xiao [56]
Fermented cow's and goat's milk	His, Tyr, Cad, Put, Spd	Solvent extraction	ODS2	Benzoyl-Cl/DAD	Costa et al. [57]
Beer	His, Cad, Tryp, Tyr, Spr	UA-DLLME	C18	BCEC-Cl/FLD	Lazaro et al. [58]
Wine	His, Tyr, Put, Cad, Spr, Det, Dmet, Prop, Agm, Spd	Dilution with water	C18	*p*-Toluenesulfonyl chloride/MS/MS	Nalazek-Rudnicka and Wasik [59]
Wine	His, Tyr, Cad, Hex, Spr, 2-pe	UA-DLLME	C18	BCEC-Cl/FLD	Wu et al. [52]
Milk	His, Put, Spd, Spr, Agm, Cad, Tyr, Trp, Phm	SSA	C18	OPA/Fluorescence (ex: 340 nm; em:445 nm)	Gloria et al. [60]

Biogenic amine: agmatine (Agm), amylamine (Am), butylamine (But), cadaverine (Cad), diethylamine (Det), dimethylamine (Dmet), dopamine (Dop), ethylamine (Et), ethanolamine (Eth), heptylamine (Hep), 1,6-hexamethylenediamine (Hex), histamine (His), isoamylamine (Iam), isobutyamine (Ibut), methylamine (Met), nitrosamine (Ntr), 2-phenylethylamine (Phm/2-PE), piperidine (Pip), propylamine (Prop), putrescine (Put), spermine (Spr), spermidine (Spd), tryptamine (Trm/Tryp), and tyramine (Tyr). Extraction: direct immersion solid-phase microextraction (DI-SPME), dispersive liquid-liquid microextraction (DLLME), matrix solid-phase dispersion (MPSD), methanesulfonic acid (MSA), perchloric acid (HClO_4_), sulphosalicylic acid (SSA), solid-phase extraction (SPE), trichloroacetic acid (TCA), ultrasound-assisted dispersive liquid-liquid microextraction (UA-DLLME), and ultrasound-assisted liquid-liquid microextraction (UA-LLE). Derivatize agent: benzoyl chloride (Bnz-Cl) (N, O-bis (trimethylsilyl) acetamide and trimethlchlorosilane) (BSA + TMCS), 2-(11H-benzo[a]carbazol-11-yl) ethyl carbonochloridate (BCEC-Cl), dabsyl chloride (Dbs-Cl), dansyl chloride (Dns-Cl), heptafluorobutyric anhydride (HFBA), isobutyl chloroformate (IBCF), o-orthophthalaldeyde (OPA), and sodium dodecylbenzenesulfonate (SDBS). Chromatography/detectors: atmospheric-pressure chemical ionization (APCI), diode-array detection (DAD), electrospray ionization (ESI), evaporative light scattering detector (ELSD), fluorescence detection (FLD), mass spectrometry (MS), and quadrupole time-of-flight (QToF).

**Table 3:** Various derivatizing agents for gas chromatography purposes and their characteristics [65–67].

No.	Reagent	Chemical structure	Characteristics
1	Acylation		
	*α*-Methoxy-*α*-trifluoromethylphenylacetic acid (MTPA)	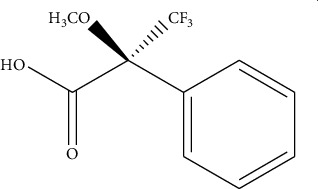	(i) It is acidic and called Mosher's acid
			(ii) Very effective in generating ions of analytes
	Acetic anhydride	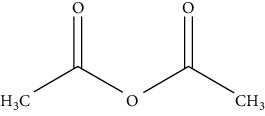	(i) Improves selectivity in GC, MS, and FID detectors, especially negative chemical ionization
			(ii) Generally used by base compounds such as biogenic amines
	4-Carbethoxyhexafluorobutyryl chloride	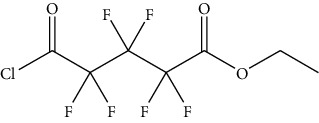	(i) Very stable for secondary amines because it can remove the excess agent by adding protic solvents
	Heptafluorobutyrylimidizole (HFBI)	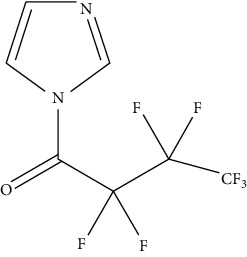	(i) Fast reaction and suitable for amine and alcohol
			(ii) Byproduct is not acidic
	*N*-Methyl-N-bis (trifluoroacetamide) (MBFTA)	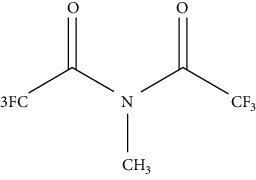	(i) Acceptable and fast for primary and secondary amines
			(ii) Byproduct is volatile
	N-(Trifluoroacetyl)-prolyl chloride (TPC)	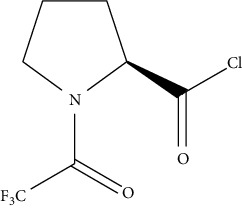	(i) Generally applied for amine compounds
			(ii) With a proton at the chiral center in the *α* position to the carbonyl group in order to avoid racemization through keto-enol tautomerization and thus reaction and storage conditions must be controlled
	Pentafluorobenzoyl chloride (PFBCI)	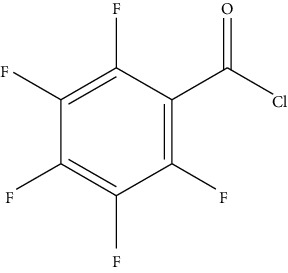	(i) Very reactive, obtaining the most sensitive ECD derivatives of amine
	Propyl chloroformate	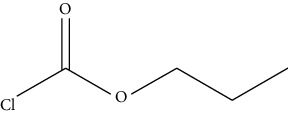	(i) Derivative analyte is water soluble; thus, the byproducts can be removed using water

2	Alkylation		
	BF_3_/methanol (*n*-butanol)	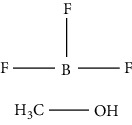	(i) Obtaining methyl (butyl) ester with acid
			(ii) Fast and quantitative esterification/transesterification
			(iii) No side reactions with volatile byproducts
	Tetrabutylammonium hydroxide (TBH)	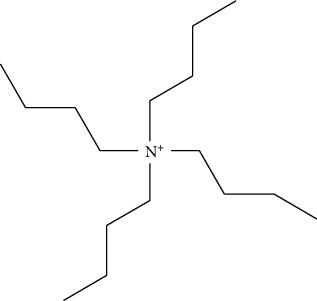	(i) Very reactive to low molecular weight amines
	Trimethylanilinium hydroxide (TMAH)	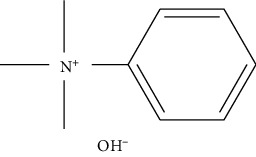	(i) Knows as a flash alkylation reagent
			(ii) Quantitative derivatization of nitrogen-bearing molecules

3	Silylation		
	Hexamethyldisilazane (HMDS)	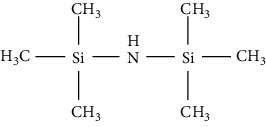	(i) A weak (trimethylsilyl) TMS donor
			(ii) It applies without solvent, yet the reagent capacity can be increased by the acidic catalyst
			(iii) Byproduct can leave the reaction mixture as the reaction goes to completion
	*N, O*-Bis (trimethylsilyl) acetamide (BSA)	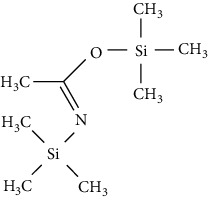	(i) Product derivatives are stable
			(ii) Byproduct elutes with the analyte
			(iii) Reactions are fast and quantitative
			(iv) BSA and its byproducts are more volatile than other silylating reagents causing less chromatographic interference
	*N, O*-Bis (trimethylsilyl) trifluoroacetamide (BSTFA)	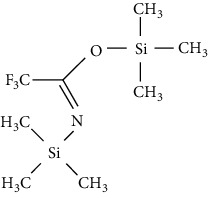	(i) Faster than BSA and more complete
			(ii) Reacts with a range of polar organic compounds by replacing active hydrogens with a TMS group
			(iii) TMCS addition is recommended in order to control hydroxyl presence and other functionalities
			(iv) Reduces the sensitivity of FID detector
	*N*-Methyl-N-(t-butyldimethylsilyl) trifluoroacetamide	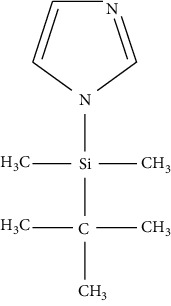	(i) Very reactive and stable
			(ii) To hinder alcohol and amine groups and add a catalyst such as *t*-butyldimethylchlorosilane
			(iii) Does not release HCl
			(iv) Very suitable for mass spectrometry detector as it provides high-mass ions
	Trimethylchlorosilane (TMCS)	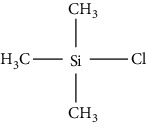	(i) A catalyst and forms HCl as a byproduct
			(ii) Completes the derivatization step after secondary amines derivatized by BSTFA by adding 1–20% TMCS to BSTFA
	Trimethylsilyldiethylamine (TMS-DEA)	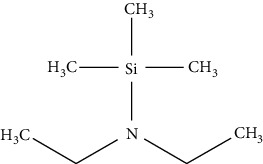	(i) Suitable for amino and carboxylic acids
	*N-*Trimethylsilylimidazole (TMSI)	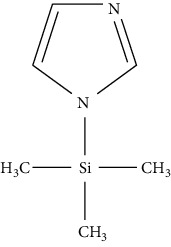	(i) Reacts with -OH and cannot react with aliphatic amines
			(ii) It is stable thermally yet more susceptible to hydrolysis

**Table 4:** Various studies based on GC techniques for biogenic amine detection in food and beverages.

Food sample	Analyte	Extraction	Stationary phase	Derivatizing/detector	Ref.
Fish and fish products	His, Tyr, Cad, Put, Spd	Distilled water	GC (column: HP-5 PMS)	BSA/FID and MS	Munir et al. [26]
Baby food products	Spd, Spm	1 M HCl	GC (column: DB-5 MS)	HFBA/MS	Petrarca et al. [70]
Canned fish	His, Tyr	5% TCA	GC (column: HP-5 MS)	SDBS/MS	Alizadeh et al. [2]
Canned fish	His, Tyr, Put, Cad	DI-SPME	GC (column: RXi-5 MS)	IBCF/MS	Huang et al. [71]
Salted fish	Ntr	Solvent extraction	GC (column: DB-Waxetr)	No derivatizing/MS-MS	Qiu et al. [72]
Cheese	His, Tyr, Put, Cad	DLLME	GC (column: HP-5 MS)	IBCF/MS	Mohammadi et al. [73]
Grape juice and wine	His, Tyr, Put, Cad, Hex, Am, Prop, But	DLLME	GC (column: HP-5 MS)	IBCF/MS	Cunha et al. [74]
Wine	His, Hex, Cad, But, Det, Put, Try, Tyr, Ibut	DI-SPME	GC (column: ZB-5 MS)	IBCF/MS	Papageorgiou et al. [75]
Wine	His, Cad, Det, Tryp, Tyr, Put, Spr, Met, Et	DLLME	GC (column: ZB-5 MS)	IBCF/MS	Plotka-Wasylka et al. [76]
Beer	His, Cad, Put, Tyr, Met, Et, Dmet, Ibut	DLLME	GC (column: DB-5 MS)	IBCF/MS	Almeida et al. [27]

Biogenic amine: amylamine (Am), butylamine (But), cadaverine (Cad), diethylamine (Det), dimethylamine (Dmet), ethylamine (Et), ethanolamine (Eth), heptylamine (Hep), 1,6-hexamethylenediamine (Hex), histamine (His), isoamylamine (Iam), isobutylamine (Ibut), methylamine (Met), nitrosamine (Ntr), 2-phenylethylamine (Phm/2-PE), piperidine (Pip), propylamine (Prop), putrescine (Put), spermine (Spr), spermidine (Spd), tryptamine (Trm/Tryp), and tyramine (Tyr). Extraction: direct immersion solid-phase microextraction (DI-SPME), dispersive liquid-liquid microextraction (DLLME), hydrochloric acid (HCl), and trichloroacetic acid (TCA). Derivatize agent: (N, O-bis (trimethylsilyl) acetamide and trimethlchlorosilane) (BSA + TMCS), heptafluorobutyric anhydride (HFBA), isobutyl chloroformate (IBCF), and sodium dodecylbenzenesulfonate (SDBS). Chromatography/detectors: Flame ionization detector (FID) and mass spectrometry (MS).
